# XPO1 inhibition sensitises CLL cells to NK cell mediated cytotoxicity and overcomes HLA-E expression

**DOI:** 10.1038/s41375-023-01984-z

**Published:** 2023-08-01

**Authors:** Jack G. Fisher, Amber D. P. Doyle, Lara V. Graham, Shreyanshi Sonar, Ben Sale, Isla Henderson, Luis Del Rio, Peter W. M. Johnson, Yosef Landesman, Mark S. Cragg, Francesco Forconi, Christopher J. Walker, Salim. I. Khakoo, Matthew D. Blunt

**Affiliations:** 1 School of Clinical and Experimental Sciences, University of Southampton, Southampton, UK; 2 School of Cancer Sciences, University of Southampton, Southampton, UK; 3 Karyopharm Therapeutics, Newton, MA 02459 USA; 4 Antibody and Vaccine Group, Centre for Cancer Immunology, Faculty of Medicine, University of Southampton, Southampton, UK; 5 Haematology Department, Cancer Care Directorate, University Hospital Southampton NHS Trust, Southampton, UK

**Keywords:** Cancer immunotherapy, Immunotherapy

## Abstract

The first-in-class inhibitor of exportin-1 (XPO1) selinexor is currently under clinical investigation in combination with the BTK inhibitor ibrutinib for patients with chronic lymphocytic leukaemia (CLL) or non-Hodgkin lymphoma. Selinexor induces apoptosis of tumour cells through nuclear retention of tumour suppressor proteins and has also recently been described to modulate natural killer (NK) cell and T cell cytotoxicity against lymphoma cells. Here, we demonstrate that XPO1 inhibition enhances NK cell effector function against primary CLL cells via downregulation of HLA-E and upregulation of TRAIL death receptors DR4 and DR5. Furthermore, selinexor potentiates NK cell activation against CLL cells in combination with several approved treatments; acalabrutinib, rituximab and obinutuzumab. We further demonstrate that lymph node associated signals (IL-4 + CD40L) inhibit NK cell activation against CLL cells via upregulation of HLA-E, and that inhibition of XPO1 can overcome this protective effect. These findings allow for the design of more efficacious combination strategies to harness NK cell effector functions against CLL.

## Introduction

The first-in-class exportin-1 (XPO1) inhibitor selinexor is FDA approved in recurrent and refractory multiple myeloma, has received accelerated approval in diffuse large B cell lymphoma and is currently under evaluation in more than sixty clinical trials for haematological malignancies and solid tumours. XPO1 is a nuclear export protein which controls the movement of proteins containing a leucine-rich nuclear export signal sequence from the nucleus to the cytoplasm [1]. It promotes the nuclear export of multiple tumour suppressor proteins, including p53, and thereby causes their inactivation [2, 3]. In addition, protein translation is critically dependent on XPO1-mediated export of ribosomal constituents [4, 5]. As a result of the pro-survival and proliferative properties of XPO1, it has a critical role in the maintenance of cancer cell survival and is frequently overexpressed in cancer [6]. Pharmacological inhibition of XPO1 leads to blockade of protein synthesis, nuclear retention of tumour suppressor proteins and apoptosis of cancer cells [4–6]. In chronic lymphocytic leukaemia (CLL) cells, XPO1 is overexpressed compared to normal B cells and *XPO1* mutations are associated with high-risk genetic abnormalities and accelerated CLL progression [7, 8]. In addition, in the Eμ-TCL1xMyc murine model of aggressive concurrent lymphoma and CLL, XPO1 inhibition provided lasting disease control [9]. The combination of selinexor and ibrutinib (NCT02303392) was tolerable and associated with clinical responses in heavily pre-treated CLL patients, including those with *BTK* mutations and Richter’s transformation [10], however further strategies are required however to improve the depth of response.

XPO1 inhibition has recently been shown to activate natural killer (NK) cells that express the inhibitory receptor NKG2A against B cell lymphoma cell lines via downregulation of HLA-E [11]. NK cells are innate lymphocytes which induce direct cytotoxicity against tumour cells via release of cytotoxic granules and death receptor signalling. NK cells can also promote immune responses through secretion of chemokines and cytokines that are crucial for the maturation and recruitment of dendritic cells, as well as for CD8 + T cell activation [12]. CLL is associated with profound immunosuppression and increased susceptibility to infectious diseases [13]. CLL patients show a significant decrease in NK cell frequency in both the peripheral blood and lymph nodes [14] whilst NK dysfunction has been associated with upregulation of the NK cell inhibitory ligand HLA-E [15, 16], shedding of NK cell activating ligands [17, 18] and increased numbers of regulatory T cells and myeloid-derived suppressor cells [19].

Through their expression of CD16A, NK cells mediate antibody-dependent cellular cytotoxicity (ADCC) and thus contribute to the efficacy of anti-CD20 monoclonal antibody (mAb) therapies including rituximab and obinutuzumab [20, 21]. Due to their anti-cancer functions, a variety of strategies designed to enhance NK cell activity against B cell malignancies are currently under clinical development [19]. These include CAR-NK cells [22], NK cell engagers [23] and adoptive transfer of ex vivo activated NK cells both alone [24, 25], and in combination with obinutuzumab and venetoclax [26]. In this study, we investigated the potential for XPO1 inhibitors to modulate NK cell activation against CLL cells in combination with BTK inhibitors and anti-CD20 antibodies, as well in the presence of microenvironmental signals that mimic lymph node support.

## Methods

### Patient CLL samples and healthy donor PBMC

Healthy donor peripheral blood mononuclear cells (PBMC) were obtained from volunteers with ethical approval from the National Research Ethics Committee (reference 06/Q1701/120) after written informed consent. PBMC were collected from 32 patients with CLL (Supplementary Table 1) recruited in the ‘real-world’ observational study at the University of Southampton (NIHR/UKCRN ID: 31076, CI F.Forconi). Diagnosis of CLL was according to the 2008 International Workshop on CLL (IWCLL2008)/National Cancer Institute (NCI) criteria [27]. Diagnosis was confirmed by a flow cytometry ‘Matutes score’ >3 in all cases. Phenotypic, immunogenetic (tumour *IGHV* usage and mutational status), FISH characteristics according to Dohner classification and *TP53* mutational status were determined, as previously described [28, 29]. CD56 + CD3- NK cells were isolated from healthy PBMCs using the Miltenyi human NK cell isolation kit and cultured in R10 medium (RPMI 1640 [Gibco] with 1% penicillin–streptomycin [Life Technologies] and 10% heat inactivated FBS [Sigma] and incubated with 1 ng/mL IL-15 (R&D Systems) overnight before use in functional assays.

### NK cell specific lysis assay

CLL cells were stained with Cell Trace™ Violet Cell Proliferation Kit (Invitrogen™) then treated for 24-hours with selinexor (Karyopharm Therapeutics), leptomycin B (Sigma), ibrutinib (Selleckchem) or acalabrutinib (Selleckchem). CLL cells were treated with the caspase inhibitor Q-VD-OPh (QVD, 30 µM, Sigma) for 30 min prior to addition of anti-cancer agents. CLL cells were then co-cultured with healthy NK cells at an effector:target (E:T) ratio of either 1:1 or 5:1 for 4 h at 37 °C. After co-culture, cells were stained with propidium iodide (Invitrogen™) and NK cell-specific lysis of CLL cells assessed on a BD FACS Aria II (BD Biosciences) using FACSDiva software (BD Biosciences) and analysed with FlowJo v10.7.1 (BD Biosciences). NK cell-specific Lysis was calculated as follows:

$$NK\;specific\;lysis = \frac{{\left( {{{{{{{{\mathrm{\% CLL}}}}}}}}\;{{{{{{{\mathrm{lysis}}}}}}}}\;{{{{{{{\mathrm{in}}}}}}}}\;{{{{{{{\mathrm{coculture}}}}}}}}} \right) - \left( {{{{{{{{\mathrm{\% spontaneous}}}}}}}}\;{{{{{{{\mathrm{CLL}}}}}}}}\;{{{{{{{\mathrm{lysis}}}}}}}}} \right)}}{{{{{{{{{\mathrm{Maximum}}}}}}}}\;{{{{{{{\mathrm{lysis}}}}}}}} - \% {{{{{{{\mathrm{spontaneous}}}}}}}}\;{{{{{{{\mathrm{CLL}}}}}}}}\;{{{{{{{\mathrm{lysis}}}}}}}}}} \ast 100$$

### Assessment of NK cell degranulation and IFNγ expression

Healthy donor PBMC were incubated with 1 ng/mL IL-15 overnight and subsequently co-cultured with selinexor- or leptomycin B-treated CLL cells at an E:T ratio of 1:1 for 4 h at 37 °C. 0.17 µg/mL α-CD107a-eFluor660 (eBioH4A3, Invitrogen) was added to PBMCs and golgiStop (BD Biosciences) added after 1 h. PBMC were then stained with the following antibodies in FACS buffer (PBS, BSA 1%, Sodium Azide 0.05%) at 4 °C for 30 min: CD3-PerCP (UCHT1, Biolegend), CD56-PE/Cy7 (hCD56, Biolegend) and NKG2A-FITC (REA110, Miltenyi Biotech). Cells were then permeabilized and fixed with BD Cytofix/Cytoperm (BD Biosciences), stained with anti-IFNγ-PE (B27, Biolegend) and assessed using a BD FACS Aria II.

### Expression of NK cell ligands

After treatment with selinexor, leptomycin B or brefeldin A (BFA, GolgiPlug, BD Bioscience) for the required timepoint, Fc receptors were blocked on CLL cells with 10% human serum for 15 min then cells were stained with CD19-PE (HIB19, Biolegend) or CD19-PB, CD5-PerCP (UCHT2, Biolegend) or CD5-APC, ULBP-1-PE (170818, R&D Systems), ULBP-2/5/6-PerCP (165903, R&D Systems), CD54-PB (HCD54, Biolegend), B7H6-APC (875001, R&D Systems), ecto-calreticulin-A488 (EPR3924, Abcam), MICA/B-PE/Cy7 (6D4, Biolegend), CD20-PE (2H7, BioLegend), CD38-A488 (HIT2, BioLegend), FAS-FITC (DX2, Biolegend), DR4-PE (DJR1, Biolegend), DR5-APC (DJR2-4, Biolegend), HLA-E-PE/Cy7 (3D12, Biolegend) and total HLA class I proteins (W6/32, Biolegend) for 30 min at 4 °C. Assessment of CLL cells that have recently egressed from the lymph nodes was performed as previously described [30, 31]. Patient CLL cells were rested for 1 h at 37 °C, then stained with CD19-BV510 (HIB19), CD5-APC, HLA-E-PE/Cy7 (3D12), pan-HLA (W6/32) and CXCR4-PE (12G5, Biolegend). Cells were then acquired on a BD FACS Aria II.

### Microenvironmental support experiments

CLL cells were stimulated with CD40L (300 ng/mL, R&D Systems), IL-4 (10 ng/mL, R&D Systems) or plate-immobilised anti-IgM F(ab’)_2_ fragments (Southern Biotechnology) 1 h prior to the addition of selinexor for a further 24 h. CLL cells were then either assessed for expression of surface ligands or co-cultured with PBMC and NK cell activation assessed by IFNγ production and CD107a expression as described above.

### TRAIL blockade

Prior to co-culture of purified NK cells or healthy donor PBMC with selinexor-treated CLL cells, NK/PBMC were incubated with 10 µg/mL anti-TRAIL (RIK-2, Biolegend, 10 µg/mL) antibodies or isotype control for 20 min at 37 °C. NK cell mediated lysis of CLL cells and NK cell degranulation was then assessed as described above.

### Assessment of NK cell activation in combination with anti-CD20 or anti-CD38 antibodies

CLL cells incubated with selinexor for 24 h were incubated with mAb (0.1 µg/mL; in-house) against CD20 (rituximab or obinutuzumab), CD38 (daratumumab) or isotype control for 20 min at 37 °C. Subsequently, CLL cells were co-cultured with healthy donor PBMC at an E:T of 1:1 for 4 h and activation assessed using the degranulation assay above.

### Immunoblotting

After 24-h treatment with selinexor, LMB or BFA, CLL cells were assessed by immunoblotting as previously described [11] using antibodies against XPO1 (D6V7N, Cell Signalling Technology), HLA-E (Sigma), BiP (3177, Cell Signalling Technology), LC3A/B (12741, Cell Signalling Technology) or β-actin (8H10D10, Cell Signalling). Protein bands were visualised using the ChemiDoc-It imaging system (UVP).

### Statistical analysis

Statistical analyses were performed using GraphPad Prism (v9.0.2) software and normal distribution of the data assessed using the Shapiro-Wilk test. One- or two-way ANOVA was used to compare differences between groups when appropriate, as described in the figure legends. A minimum of three different CLL donors were assessed for each experiment, as detailed in the figure legends. Error bars represent standard error of the mean and data were considered statistically significant at *P* < 0.05.

## Results

### XPO1 inhibition sensitises CLL cells to NK cell anti-tumour functions

**Fig. 1:**
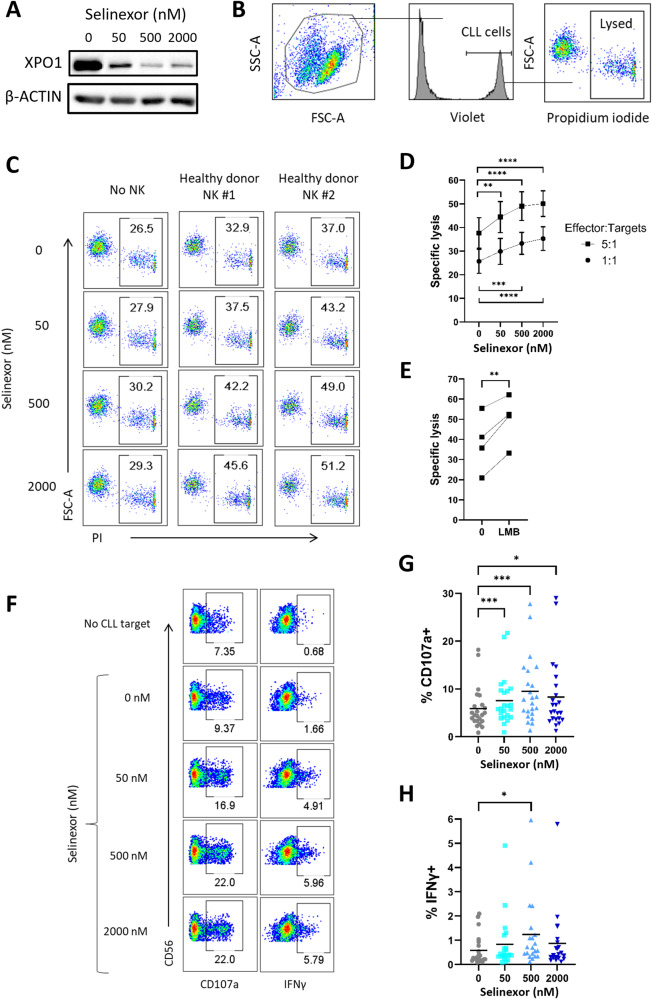
XPO1 inhibition sensitises primary CLL cells to NK cell mediated cytotoxicity. **A** CLL cells were treated with selinexor (50–2000 nM) for 24 h in the presence 30 µM Q-VD and cell lysates analysed by western blot. Shown is a representative example of three CLL donors. **B** Flow cytometry gating strategy for measuring NK specific lysis of CLL cells. Prior to 24-hour selinexor treatment, CLL cells were stained with CellTrace Violet to allow identification of CLL cells in co-culture. Propidium iodide (PI) was used to measure the proportion of lysed CLL cells. **C** Representative example of the proportion of lysed CLL cells from one CLL donor when treated with selinexor for 24 h in the presence 30 µM Q-VD (left) and then co-cultured with purified NK cells for 4 h after selinexor treatment at an E:T ratio of 5:1 (centre and right). **D**, **E** NK specific lysis of CLL target cells pre-treated for 24 h with selinexor (**D**, *N* = 10) or 50 nM leptomycin B (**E**, LMB, E:T = 5:1, *N* = 4) in the presence 30 µM Q-VD. Differences in the lysis ability of NK cells was calculated between selinexor concentrations at each E:T using repeated-measure one-way ANOVA followed by Dunnett’s multiple comparisons test (**P < 0.01, ***P < 0.005, ****P < 0.001). Paired *t* test (**P < 0.01) was used to calculate a significant difference in NK cell lysis with LMB. **F** CD56 + CD3- NK cell activation after 4 h co-culture of healthy donor PBMC with selinexor-treated CLL cells. Activation was measured as the proportion of NK cells positive for CD107a and IFNγ. Activation gates were drawn based on the no CLL target control condition and background activation subtracted from co-culture samples and used to plot graphs in (**G**) and (**H**). Percentage of CD107a+ (**G**) and IFNγ+ (**H**) NK cells after co-culture with selinexor-treated CLL cells. Line on graph represents the mean of each group (CD107a: *N* = 23 and IFNγ: *N* = 21). Differences in NK cell activation between selinexor treatments were calculated by repeated-measure one-way ANOVA followed by Dunnett’s multiple comparison test (*P < 0.05, ***P < 0.005).

CLL cells were incubated with selinexor for 24 h at concentrations achievable in patient plasma (50–2000 nM) [32], and then either assessed for XPO1 protein expression or co-cultured with healthy donor NK cells. Selinexor induced a concentration-dependent reduction in XPO1 expression in CLL cells (Fig. 1A), in accordance with previous reports [11]. NK-mediated lysis of CLL cells was increased in a concentration dependent manner by selinexor pre-treatment at both a 5:1 (mean 37% lysis at 0 nM rising to 50% at 2000 nM, *P* < 0.001) and 1:1 (mean 25% lysis at 0nM rising to 35% at 2000nM, *P* < 0.001) effector:target ratio (Fig. 1B–D). This effect was NK cell specific as apoptosis induced directly by selinexor was prevented by addition of the caspase inhibitor QVD (Fig. 1C). To confirm that XPO1 inhibition increases NK cell activation against CLL, we tested the alternative XPO1 inhibitor leptomycin B (LMB), a *Streptomyces* metabolite. These data demonstrated that XPO1 inhibition with LMB also significantly (*P* < 0.01) increased NK cytotoxicity against CLL cells (Fig. 1E). The pre-treatment of CLL cells with selinexor also enhanced NK cell activation as measured by both degranulation (increased CD107a positivity) (mean 5.26% at 0 nM rising to 9.35% at 500 nM, *P* < 0.005) and IFNγ expression (mean 0.54% at 0 nM rising to 1.23% at 500 nM, *P* < 0.05) (Fig. 1F–H). These results demonstrate that XPO1 inhibition primes CLL cells to NK cell effector function.

### XPO1 inhibition reduces surface expression of HLA-E on CLL cells

**Fig. 2:**
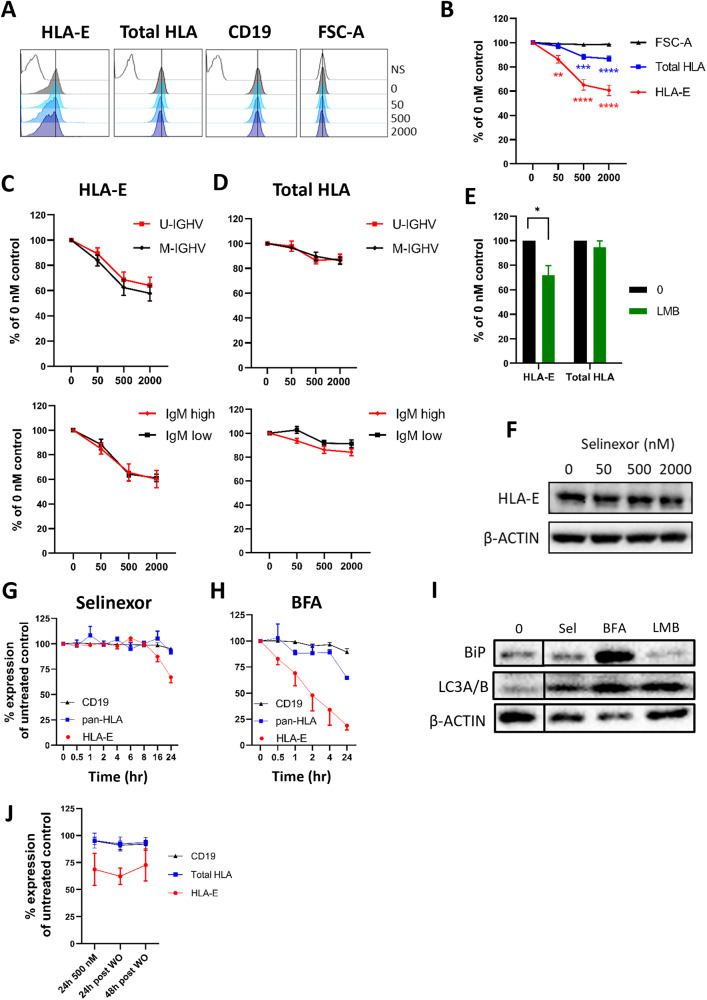
Selinexor downregulates HLA-E on the surface of CLL cells independently of IGHV mutational status. **A** Histograms of the surface expression on CD19 + CD5 + CLL cells showing HLA-E, total HLA class I or CD19 on a representative CLL donor after treatment with selinexor (50–2000 nM) for 24 h. Cell size was measured using the FSC-A parameter. NS = no stain. Black vertical line on each histogram highlights the position of the 0 nM control. **B** Surface expression of HLA-E and total HLA class I on CD19 + CD5 + CLL cells after treatment with selinexor (50–2000 nM) for 24 h. Shown is mean expression ± SEM relative to the 0 nM control (*N* = 16 CLL donors). Asterisks represent significant differences in HLA surface expression compared to the 0 nM control and was calculated with repeated-measure one-way ANOVA followed by Dunnett’s multiple comparison test (**P < 0.01, ***P < 0.005, ****P < 0.001). Surface expression of HLA-E (**C**) and total HLA class I (**D**) on CD19 + CD5 + CLL cells with unmutated (U-IGHV, *N* = 7) or mutated IGHV (M-IGHV, *N* = 9) and on donors with high (*N* = 10) or low IgM (*N* = 6) surface expression. **E** Surface expression of HLA-E and total HLA class I on CLL cells treated with leptomycin B (LMB, 50 nM) for 24 h. Shown is mean expression ± SEM relative to the 0 nM control (*N* = 4) and significant differences in surface expression to the 0 nM control were calculated with paired-sample two-way ANOVA followed by Sidak’s post-hoc test (*P < 0.05). **F** Abundance of HLA-E in CLL cells as measured by immunoblotting after treatment with selinexor for 24 h. Surface expression of HLA-E, total HLA class I molecules and CD19 on CD19 + CD5 + CLL cells after treatment with 500 nM selinexor (**G**, *N* = 6) or 2 µg/mL brefeldin A (BFA, **H**, *N* = 3) at the indicated time points. Shown is mean ± SEM. **I** Abundance of the ER stress marker BiP and the autophagy marker LC3A/B in CLL cells treated with 2000 nM selinexor (Sel), 50 nM Leptomycin B (LMB) or 2 µg/mL brefeldin A (BFA) for 24 h. **J** HLA-E, total HLA and CD19 expression on CLL cells after removal of selinexor from culture. After 24 hr treatment, selinexor was removed from the culture and surface expression of CD19 and HLA proteins were assessed on CLL cells 24 h and 48 h post selinexor wash out (WO). Shown is mean ± SEM of four CLL patient samples.

To determine the mechanism for this, we measured expression of key inhibitory molecules for NK cells expressed on CLL cells; HLA-E and total HLA (HLA-A/B/C/E). Selinexor significantly decreased surface expression of HLA-E on CLL cells (mean 42% reduction at 2000nM, *p* < 0.001) in all 16 patient samples assessed (IQR = 28% to 48% reduction at 2000 nM) (Fig. 2A, B). In contrast, the expression of total HLA class I molecules reduced by only ~10%, indicating relative preservation of classical HLA class I, HLA-A, -B, and -C. Decreased expression of HLA-E following selinexor treatment was not due to decreased cell size as the FSC-A remained constant, nor was it generally observed for other surface markers, as levels of CD19 remained stable (Fig. 2A, B). The mutational status of the immunoglobulin heavy chain (IGHV) and surface expression of IgM are associated with disease prognosis in CLL [29, 33] and we observed no difference in HLA-E downregulation between cases with mutated- or unmutated-IGHV, or with high or low surface IgM expression (Fig. 2C, D). In addition, incubation of CLL cells with leptomycin B also caused a significant (mean 36% reduction, *P* < 0.05) downregulation of HLA-E, but not total HLA, surface expression (Fig. 2E).

In contrast to surface expression, the total level of intracellular HLA-E expression remained unchanged following XPO1 inhibition (Fig. 2F). Stabilisation of surface HLA-E on myeloma cells requires the delivery of de novo synthesised molecules from the ER and HLA-E downregulation is associated with ER stress and autophagy markers(34). XPO1 inhibition inhibits protein translational activities [4, 5], and we therefore compared the ability of selinexor to downregulate HLA-E versus the ER-Golgi transport inhibitor brefaldin A (BFA). HLA-E was downregulated on CLL cells by selinexor between 16 and 24 h, whereas BFA rapidly reduced surface HLA-E expression, beginning at 30 min (Fig. 2G, H), in accordance with the direct and rapid blockade of protein transport by BFA. Selinexor did not induce expression of the ER stress marker BiP but did increase expression of the autophagy marker LC3A/B (Fig. 2I). This data indicates that CLL surface expression of HLA-E is highly sensitive to disruption of newly synthesised protein transport and is associated with the induction of autophagy markers, in accordance with previous reports [11, 34, 35].

To assess the duration of HLA-E downregulation, we performed drug washout experiments. After 24 h incubation with selinexor (500 nM), cells were either immediately analysed for surface HLA-E, total HLA and CD19 expression, or selinexor was removed and surface receptor expression analysed after a further 24 and 48 h of culture in fresh media. This showed that HLA-E remains suppressed (>25%) on the surface of CLL cells for at least 48 h post wash-out of selinexor (Fig. 2J). These data demonstrate that XPO1 inhibition causes prolonged and selective downregulation of surface HLA-E on CLL cells, independently of known prognostic markers.

### XPO1 inhibition enhances NKG2A+ and NKG2A− NK cell activation against CLL

**Fig. 3:**
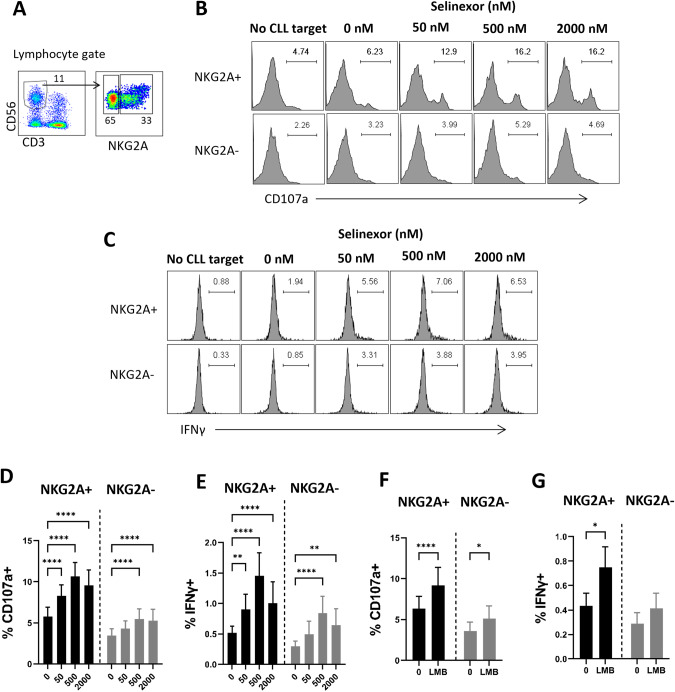
XPO1 inhibition activates NKG2A+ and NKG2A− NK cells against CLL cells. **A** Flow cytometry gating strategy to identify NKG2A+ and NKG2A- NK cells within the CD3-CD56 + NK cell population of healthy donor PBMC. Representative example of NK cell activation as measured by degranulation (CD107a staining, **B**) and IFNγ production (**C**) in NKG2A+ and NKG2A- NK cells after healthy donor PBMC were co-cultured for 4 h with CLL cells pre-treated with selinexor (50–2000 nM) for 24 h. NK cell activation measured by CD107a staining (**D**) and IFNγ production (**E**) in NKG2A+ and NKG2A- NK cells when healthy donor PBMC were co-cultured for 4 h with selinexor-treated (50–2000 nM) CLL cells. Shown is mean ± SEM (*N* = 23) and differences in NK activation compared to the untreated control were assessed by repeated-measure two-way ANOVA followed by Dunnett’s multiple comparison test (**P < 0.01, ****P < 0.001). NK cell activation measured by CD107a staining (**F**) and IFNγ production (**G**) in NKG2A+ and NKG2A- NK cells when healthy donor PBMC were co-cultured for 4 h with leptomycin-treated (LMB, 50 nM) CLL cells. Shown is mean ± SEM (*N* = 17) and differences in NK activation compared to the untreated control were assessed by repeated-measure two-way ANOVA followed by Dunnett’s multiple comparison test (*P < 0.05, ****P < 0.001).

HLA-E is the ligand for the key NK cell inhibitory receptor NKG2A, and we therefore assessed the activity of NKG2A + NK cells versus NKG2A- NK cells against selinexor-treated CLL cells (Fig. 3A). NKG2A + NK cell activity against CLL cells was significantly increased by selinexor at all concentrations tested (50–2000 nM) as measured by both degranulation (mean 5.8% at 0 nM rising to 10.6% at 500 nM, *P* < 0.001) and IFNγ expression (mean 0.5% at 0 nM rising to 1.5% at 500 nM, *P* < 0.001) (Fig. 3B–E). Consistent with the plateauing of HLA-E expression between 500 and 2000 nM (Fig. 2B), NK cell activation was increased at 500 nM selinexor and not further increased at 2000nM (Fig. 3B–E). In addition, NKG2A- NK cell activation was also increased at the higher concentrations of selinexor (500–2000 nM) (Fig. 3B–E), albeit to a lesser extent than NKG2A + NK cells. In accordance with these data, leptomycin B incubation with CLL cells increased NKG2A+ (*P* < 0.001) and NKG2A- (*P* < 0.05) NK cell degranulation (Fig. 3F), but again increases were far higher in NKG2A + NK cells. Perhaps reflecting this increased sensitivity, leptomycin B significantly (*P* < 0.05) increased IFNγ expression in NKG2A + NK cells only (Fig. 3G). These data indicate that NKG2A + NK cells are activated more readily against CLL cells in the presence of XPO1 inhibitors compared to NKG2A- NK cells, however XPO1 inhibition also activates NK cells that are negative for NKG2A, albeit to a lesser extent. The levels of NK cell activation induced in these assays in the absence of XPO1 inhibition is consistent with previous reports of low NK cell cytotoxicity against primary CLL cells [16].

**Fig. 4:**
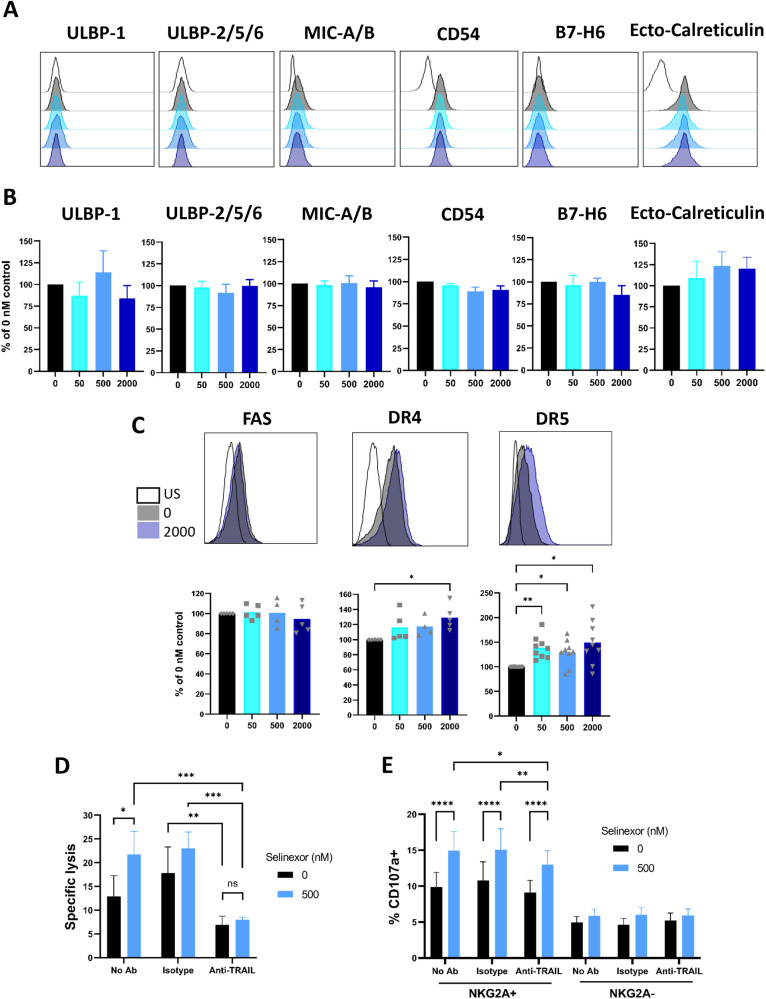
XPO1 inhibition modulates CLL cell expression of activating NK cell ligands. Expression of NK cell activating ligands ULBP-1, ULBP-2/5/6, MIC-A/B, B7H6, CD54/ICAM-1 and ecto-calreticulin on CD5 + CD19 + CLL cells after treatment with selinexor (50–2000 nM) for 24 h. Shown are histograms of a representative CLL sample (**A**) for each ligand and (**B**) mean ± SEM of multiple CLL donors (*N* = 3–7 per group). US = unstained. **C** Expression of death receptors Fas, DR4 and DR5 on CLL cells post selinexor 24-h treatment. Shown are the histograms of a representative CLL donor and below is the mean ± SEM across multiple CLL donors (Fas and DR4 *N* = 5 and DR5 *N* = 9). Differences in expression between selinexor concentrations were assessed by repeated measure one-way ANOVA followed by Dunnett’s multiple comparisons test (*P < 0.05, **P < 0.01). NK specific lysis of selinexor-treated (500 nM) CLL targets (**D**) and NKG2A+ and NKG2A- NK cell activation (**E**) in the presence of a TRAIL blockade antibody. Isolated NK cells (**D**) or healthy donor PBMC (**E**) were incubated with 10 µg/mL anti-TRAIL or isotype control antibody for 20 min prior to co-culture with selinexor-treated CLL cells. Shown is mean ± SEM (**D**: *N* = 6 and **E**: *N* = 8) and differences in NK specific lysis and NK cell activation between treatments were calculated with repeated-measure two-way ANOVA followed by Tukey’s post-hoc test (*P < 0.05, **P < 0.01, ***P < 0.005, ****P < 0.001).

To determine the mechanism for enhanced activation of NKG2A- NK cells by XPO1 inhibitors, we assessed expression of NK cell activating ligands on CLL cells. Following selinexor incubation, we observed no significant change in the surface expression of ULBP-1, ULBP-2/5/6, MIC-A/B, CD54 (ICAM-1), B7-H6, or externalised calreticulin (ecto-reticulin), recently identified as the cancer associated ligand for NKp46 [36] (Fig. 4A, B). We then assessed the effect of selinexor on CLL expression of the death receptors Fas, DR4 and DR5. No change in expression was seen for Fas, however both DR4 (29% increase, P < 0.05) and DR5 (46% increase, *P* < 0.05) were significantly increased by selinexor on the surface of CLL cells (Fig. 4C). DR4 and DR5 can engage their ligand TRAIL on NK cells to induce NK cell degranulation and IFNγ production [37] and the enhanced NK mediated lysis of CLL cells induced by selinexor (12.9% to 21.8%, *P* < 0.05) was ablated (7–8%, no statistical significance) in the presence of anti-TRAIL blocking antibody (Fig. 4D). Furthermore, TRAIL blockade partially (*p* < 0.01) reduced the degranulation of NKG2A + NK cells against CLL cells in the presence of selinexor (Fig. 4E). These data indicate that XPO1 inhibition increases DR4 and DR5 expression on CLL cells and that TRAIL interactions contribute significantly to NK-mediated lysis and activation of CLL cells.

### Selinexor enhances NK cell-mediated effector functions towards CLL cells in combination with anti-CD20 and anti-CD38 mAb

**Fig. 5:**
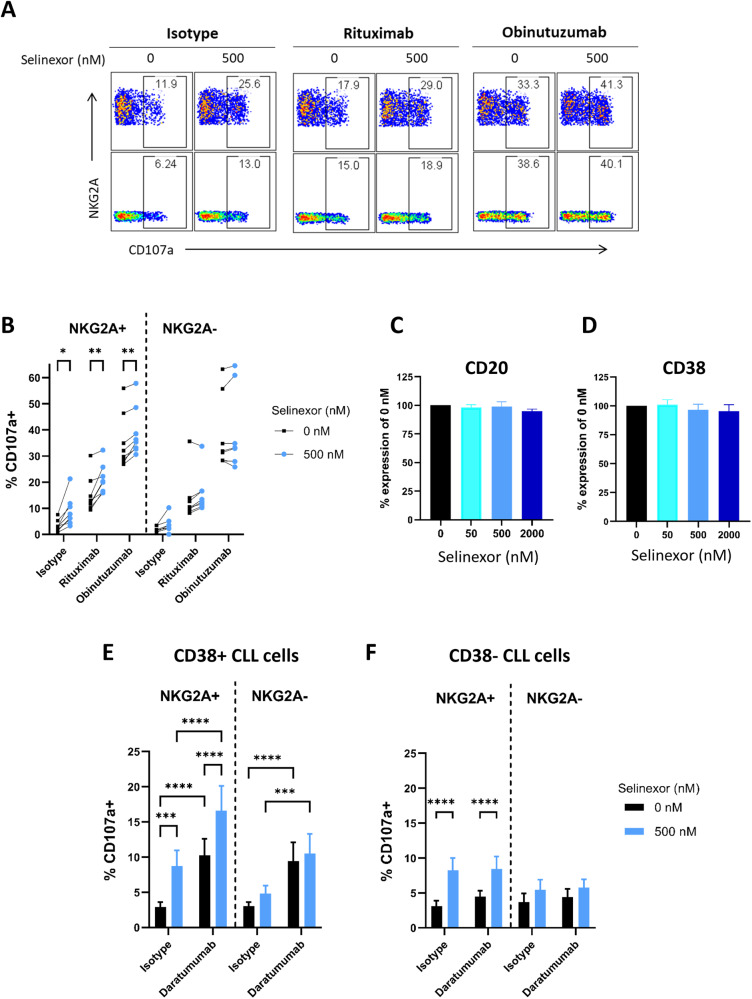
Selinexor enhances NK cell activation against CLL cells in combination with anti-CD20 and anti-CD38 mAb. Flow cytometry plots of NKG2A+ and NKG2A- NK cell activation measured by CD107a expression against CLL cells pre-treated with selinexor and anti-CD20 mAbs rituximab and obinutuzumab or isotype control (**A**). CLL cells were treated with 500 nM selinexor or DMSO for 24 h and incubated with 0.1 µg/mL anti-CD20 mAb rituximab or obinutuzumab or isotype control for 20 min prior to co-culture with healthy donor PBMC (E:T = 1:1). NK cell activation in NKG2A+ and NKG2A- NK cells was measured by CD107a staining minus the no target control for each mAb (**B**). Each line in (**B**) represents matched co-cultures for each antibody condition (*N* = 8). Significant differences in NK cell activation between selinexor concentrations were assessed by repeated-measure two-way ANOVA followed by Sidak’s multiple comparison test (*P < 0.05, **P < 0.01). **C**, **D** Expression of CD20 (**B**, *N* = 5) and CD38 (**C**, N = 4) on CD5 + CD19 + CLL cells after treatment with selinexor (50–2000 nM) for 24 h. **E**, **F** NK cell activation in NKG2A+ and NKG2A- NK cells against selinexor-treated CLL cells in the presence of daratumumab (anti-CD38). CLL cells derived from donors positive (D, *N* = 7) and negative (E, N = 11) for CD38 were treated with 500 nM selinexor for 24 h and co-cultured with healthy donor PBMC the next day (E:T = 1:1). Prior to co-culture CLL cells were incubated with daratumumab or isotype control for 20 min at a concentration of 0.1 µg/mL. Shown is the mean ± SEM and differences in NK cell activation between treatments were assessed by repeated-measure two-way ANOVA followed by Tukey’s post-hoc test (***P < 0.005, ****P < 0.001).

NK cells mediate ADCC against target cells and we therefore investigated whether selinexor could enhance NK-mediated effector functions against CLL cells in combination with mAb. Pre-treatment of CLL cells with selinexor (500 nM) significantly (*p* < 0.01) increased NKG2A + NK cell degranulation against CLL cells in combination with both rituximab and obinutuzumab in all donors assessed (*n* = 8) (Fig. 5A, B). No effect of selinexor on CLL surface expression of CD20 was evident (Fig. 5C). To test whether this effect was specific to anti-CD20 mAb or more generally applicable as a means to increase NK cell activation in combination with tumour targeting mAb, we next investigated the anti-CD38 mAb daratumumab. Selinexor did not alter the surface expression of CD38 on CLL cells (Fig. 5D) however the pre-treatment of CD38 + CLL cells with selinexor (500 nM) significantly (*p* < 0.001) increased NKG2A + NK cell degranulation in combination with daratumumab (Fig. 5E). To confirm this effect was due to the engagement of target antigen, we then assessed CLL samples with no detectable surface expression of CD38. Accordingly, although selinexor increased NK cell activation against CD38- CLL cells, there was no increase in NK cell activation by daratumumab against CD38- CLL cells (Fig. 5F). These data demonstrate that selinexor enhances NK cell activation against CLL cells in combination with tumour targeting antibodies.

### Effect of selinexor on NK cell activation in combination with BTK inhibitors

Selinexor is currently in a phase 1 trial in combination with the BTK inhibitor (BTKi) ibrutinib for patients with relapsed/refractory CLL or aggressive non-Hodgkin lymphoma (NCT02303392) [10]. This trial is of interest as ibrutinib can induce NK cell dysfunction via off-target inhibition of ITK [38, 39]. We therefore tested whether selinexor retained its capacity to enhance NK cell activity in combination ibrutinib. We also assessed whether the combination of selinexor with a more selective BTK inhibitor (acalabrutinib) with reduced inhibitory activity against ITK and NK cells [40, 41] would allow for superior NK cell function.

**Fig. 6:**
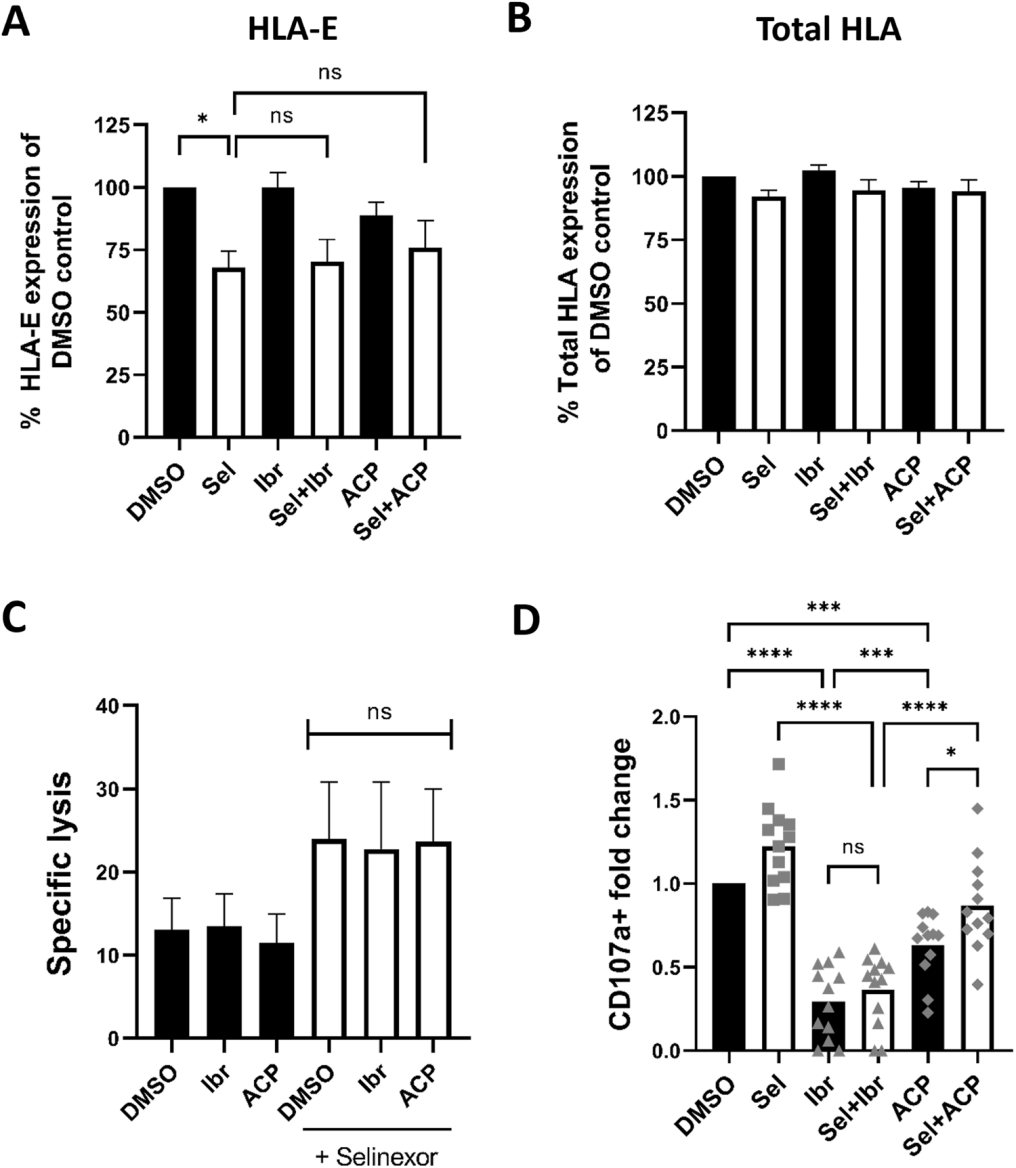
Selinexor enhances NK cell activation against CLL cells in combination with acalabrutinib. Expression of HLA-E (**A**) and total HLA class I proteins (**B**) on CD19 + CD5 + CLL cells after treatment with selinexor (500 nM) and the BTK inhibitors Ibrutinib (Ibr, 1 µM) and Acalabrutinib (ACP, 1 µM) as indicated for 24 h. Shown is mean ± SEM (*N* = 5) and significant differences in ligand expression between treatments were calculated with repeated-measure one-way ANOVA followed by Tukey’s post-hoc test (*P < 0.05). **C** NK specific lysis of CLL targets pre-treated for 24 h with selinexor (500 nM), Ibr (1 µM) or ACP (1 µM) alone or in combination as indicated (E:T = 5:1). Shown is the mean ± SEM (*N* = 4) and differences in NK cell specific lysis between combination treatments were calculated with repeated-measure one-way ANOVA. **D** NK cell activation as measured by fold change in CD107a staining when healthy donor PBMC were co-cultured with CLL cells pre-treated for 24 h with selinexor (500 nM), Ibr (1 µM) and ACP (1 µM) as indicated (E:T = 5:1). During the 4-h co-culture, selinexor and BTK inhibitors were re-added to appropriate wells. Shown is the mean ± SEM (*N* = 12) and differences in NK cell activation between treatments were assessed by repeated-measure one-ANOVA followed by Tukey’s post-hoc test (*P < 0.05, ***P < 0.005, ****P < 0.001). ns nonsignificant.

We observed significant (*P* < 0.05) HLA-E downregulation in selinexor but not BTKi-only treated samples (Fig. 6A). Importantly, the combination of either BTKi with selinexor showed no significant effect on selinexor mediated downregulation of HLA-E (Fig. 6A). In addition, selinexor, ibrutinib and acalabrutinib did not significantly alter levels of total HLA (Fig. 6B). Consequently, we assessed the ability of NK cells to lyse CLL targets that were pre-treated with these drug combinations, with drugs washed out for the 4 h NK:CLL co-culture. Consistent with the data on HLA-E expression, neither ibrutinib nor acalabrutinib pre-treatment impeded the enhanced NK activation evident with selinexor (Fig. 6C). In addition, pre-incubation of CLL cells with BTKi alone did not affect NK cell specific lysis as compared to the DMSO control (Fig. 6C).

Subsequently, CLL cells were pre-treated with the indicated drug combinations for 24-h then co-cultured with NK cells for 4 h with the corresponding drug combinations added back in during the co-culture. In the presence of ibrutinib, NK cell degranulation was significantly diminished compared to selinexor alone and the addition of selinexor was unable to overcome this effect (mean fold change 1.2 vs 0.36, *P* < 0.001) (Fig. 6D). In contrast, the combination of acalabrutinib with selinexor showed significantly higher NK cell degranulation compared to ibrutinib + selinexor (mean fold change of 0.87 vs 0.36, *P* < 0.001) and acalabrutinib alone (mean fold change 0.87 vs 0.63, *P* < 0.05) (Fig. 6D). Compared to DMSO control, acalabrutinib alone significantly inhibited NK cell activation (mean fold change 0.63, *P* < 0.005), however this was to a lesser extent than ibrutinib alone (mean fold change 0.29, *P* < 0.001), in accordance with previous reports [42]. These data suggest that the combination of selinexor with a more specific BTK inhibitor allows for enhanced NK cell activity against CLL cells.

### Lymph node associated signals inhibit NK cell activity against CLL cells via NKG2A, and are overcome by XPO1 inhibition

CLL tumour cells receive supportive microenvironmental signals, such as BCR ligation, IL-4 and CD40L, within the lymph nodes of patients, promoting tumour cell survival, proliferation and drug resistance [43–45]. These contributions are thought to impact significantly on prognosis and treatment success and so we assessed whether the signals that CLL cells can receive in the lymph nodes could also modulate the sensitivity of CLL cells to NK mediated effector functions.

**Fig. 7:**
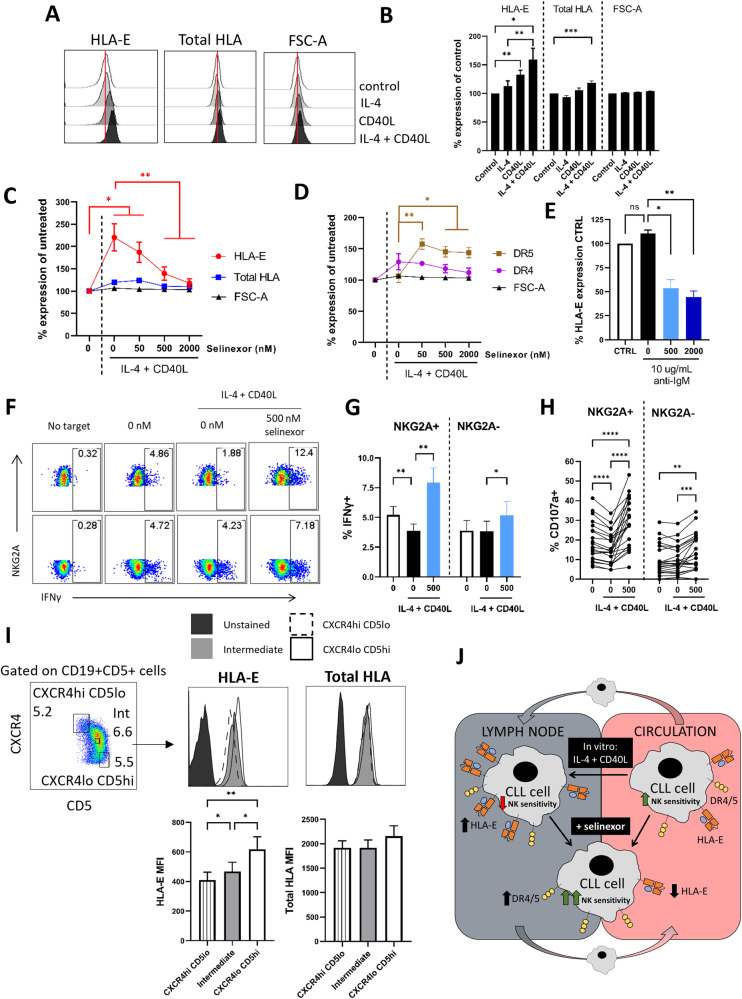
IL-4 + CD40L stimulation inhibits NK cell activity against CLL cells, which is overcome by XPO1 inhibition. Expression of HLA-E and HLA class I molecules (Total HLA) on CD19 + CD5 + CLL cells after incubation with IL-4 (10 ng/mL) and CD40L (300 ng/mL) alone or in combination for 24 h. Representative histograms from one CLL sample is shown in (**A**). Cell size was assessed by FSC-A. Shown in (**B**) is the mean ± SEM (*N* = 18) percentage expression of HLA-E or total-HLA relative to the untreated control. Differences in expression between conditions were assessed by repeated-measure two-way ANOVA followed by Tukey’s post-hoc test (*P < 0.05, **P < 0.01, ***P < 0.005). Expression of HLA-E and total HLA class I (**C**) and DR4 and DR5 (**D**) on CD19 + CD5 + CLL cells after treatment with selinexor (50–2000 nM) for 24 h in the presence of IL-4 (10 ng/mL) and CD40L (300 ng/mL). Shown is the mean ± SEM of 12 CLL patient samples and differences in expression between conditions were assessed by repeated-measure two-way ANOVA followed by Tukey’s post-hoc test (*P < 0.05, **P < 0.01). **E** HLA-E expression on CLL cells treated with selinexor following BCR stimulation. CLL cells were incubated with 10 µg/mL plate-immobilised anti-IgM F(ab’)_2_ fragments for 1 h then selinexor (500–2000 nM) or DMSO (0 nM) was added for a further 24 h before assessment of HLA-E expression. Shown is mean ± SEM relative to the untreated control (*N* = 3) and differences in expression between treatments were calculated by repeated-measure one-way ANOVA followed by Tukey’s post-hoc test (*P < 0.05, **P < 0.01). NK cell activity measured by expression of IFNγ (**F**, **G**) and CD107a (**H**) in NKG2A+ and NKG2A- NK cells when heathy donor PBMC were co-cultured for 4 h with CLL cells (E:T = 1:1). CLL cells were pre-treated with 500 nM selinexor or DMSO (0 nM) in the presence of IL-4 (10 ng/mL) and CD40L (300 ng/mL) for 24 h. A representative example for IFNγ expression is shown in (**F**). Graph in (**G**) shows mean ± SEM (*N* = 19) and in (**H**) each individual co-culture is represented by a dot (*N* = 22 per condition). Significant differences in NK activation between conditions were assessed by repeated-measure two-way ANOVA followed by Tukey’s post-hoc test (**P < 0.01, ***P < 0.005, ****P < 0.001). **I** HLA-E and total HLA expression on CLL cell populations with differential expression of CXCR4 and CD5. Shown is the gating strategy used to identify CXCR4hi(high) CD5lo(low) and CXCR4lo CD5hi CLL cells and CLL cells with intermediate expression of CXCR4 and CD5. For each population, gates were drawn around ~5–7% of total CD19 + CD5 + CLL cells and HLA-E and total HLA expression was assessed as shown above for one representative CLL donor. Below is the summary (mean ± SEM) of the geometric MFI (MFI) for HLA-E and total HLA across 10 different CLL donors. Differences in expression between CLL cell populations were calculated using repeated-measure one-way ANOVA followed by Tukey’s post-hoc test (*P < 0.05, **P < 0.01). **J** Schematic diagram for the modulation of CLL cell sensitivity to NK cell cytotoxicity by lymph node associated signals and selinexor. The lymph node associated signals IL-4 and CD40L upregulate surface HLA-E on CLL cells, and this inhibits NK cell effector function via NKG2A. Selinexor reduces HLA-E expression on CLL cells in both the absence and presence of IL-4 + CD40L and thereby increases the sensitivity of CLL cells to NK cell activity. In addition, selinexor increases expression of the TRAIL death receptors DR4 and DR5 on CLL cells and this contributes to the enhanced NK cell activation against CLL cells.

Incubation of CLL cells with CD40L significantly increased surface HLA-E expression (*P* < 0.01), with no effect on total HLA expression (Fig. 7A, B). The combination of IL-4 and CD40L together led to a further increase in HLA-E expression on CLL cells (mean IL-4 + CD40L 160% (*P* < 0.05), IL-4 121% and CD40L 132%), and increased total HLA to a lesser extent (118%, *P* < 0.001) (Fig. 7A, B). To assess whether selinexor could overcome this effect, we incubated CLL cells with IL-4 + CD40L 1 h prior to incubation with selinexor for a further 24 h and then assessed HLA-E. Selinexor (500–2000 nM) significantly reduced HLA-E expression in the presence of IL-4 + CD40L and abolished the effect of IL-4 + CD40L on HLA-E expression at 2000nM (*P* < 0.01) (Fig. 7C). In addition, increased expression of the death receptor DR5 was evident following selinexor treatment in the presence of IL-4 + CD40L (Fig. 7D). In contrast to IL-4 + CD40L, anti-IgM stimulation showed no significant alteration to the expression of surface HLA-E on CLL cells (Fig. 7E). Together, these data indicate that signals present in the lymph node microenvironment can alter expression of NK cell ligands on CLL cells. Therefore, we tested whether NK cell activation was also altered under these conditions. Incubation of CLL cells with IL-4 + CD40L significantly reduced NKG2A+, but not NKG2A-, NK cell activation against CLL cells as measured by IFNγ production (mean 5.2–3.8%, *P* < 0.01) and degranulation (mean 18.7–15.9%, *P* < 0.001) (Fig. 7F–H), consistent with NKG2A acting as an inhibitory immune checkpoint [46]. In the presence of IL-4 + CD40L support, selinexor (500 nM) significantly increased IFNγ production and degranulation against CLL cells in both NKG2A+ and NKG2A- NK populations (Fig. 7F–H).

To investigate whether CLL expression of HLA-E in patients is altered by the lymph node microenvironment, we utilised a previously reported gating strategy whereby CXCR4low CD5high expression defines a population of CLL cells that have recently egressed from the lymph nodes into the blood [31]. Consistent with our in vitro data, CXCR4low CD5high CLL cells that have recently egressed from the lymph nodes expressed significantly higher levels of surface HLA-E compared to CLL cells that have been in the circulation for longer; defined as CXCR4high CD5low and CXCR4intermediate CD5intermediate (Fig. 7I). These data demonstrate that lymph node associated signals impair NK cell activity against CLL cells, and that inhibiting XPO1 can overcome this effect.

## Discussion

The mechanisms behind NK cell dysfunction in CLL are poorly understood and restoration of NK cell function may allow for improved anti-tumour and anti-infection immunity in patients. In this study, we identify that the first-in-class XPO1 inhibitor selinexor potentiates NK cell activity against CLL cells alone and in combination with acalabrutinib and anti-CD20 mAb via downregulation of HLA-E and upregulation of TRAIL death receptors DR4 and DR5. In addition, we identify that lymph node associated signals (IL-4 + CD40L) increase HLA-E expression on the surface on CLL cells and that this inhibits NK cells via NKG2A. Importantly, we show that inhibition of XPO1 can overcome this protective effect and potentiate NK effector functions (Fig. 7J).

Compared to healthy controls, NK cells in CLL patients demonstrate reduced frequency in the blood and lymph nodes [14], decreased degranulation [47], cytotoxicity [48] and ADCC [49], downregulation of activating receptors [48] and increased CLL surface expression of HLA-E [15, 18]. Our study adds to this literature, with the identification that lymph node associated signals inhibit NK cell activation against CLL cells via the NKG2A:HLA-E immune checkpoint axis. IL-4 signalling is enriched predominantly in the lymph nodes [45] whereas T cells positive for CD40L are found in both the lymph nodes and bone marrow of CLL patients [50]. HLA-E expression on CLL cells therefore may be modulated by microenvironmental signalling within both the lymph nodes and the bone marrow in patients. No difference in NKG2A expression is evident on NK cells in CLL compared to healthy controls, however NKG2A blockade improves CLL patient NK cell function [15].

The first-in-class XPO1 inhibitor selinexor downregulated surface HLA-E expression on all CLL samples tested and led to enhanced NK cell lysis of CLL cells and IFNγ production. This suggests that selinexor could potentially be used to prime malignant B cells for NK cell therapies. Indeed, enhanced NK cell activity against CLL cells was evident with selinexor pre-treatment in combination with the anti-CD20 mAb rituximab and obinutuzumab. B cell lymphoma patients with lower NK numbers have shorter progression free survival during anti-CD20 treatment [20] and hence strategies which promote NK cell function to potentially compensate for this are of high interest. A recent phase I trial showed that selinexor in combination with ibrutinib induced clinical responses and had an acceptable toxicity profile in heavily pre-treated CLL patients [10]. Our study indicates that the combination of selinexor with more selective BTK inhibitors, such as acalabrutinib, could potentially provide better efficacy, in line with previous reports showing that ibrutinib inhibits NK cell function through inhibition of ITK [38, 42, 51, 52] whereas acalabrutinib is less disruptive to NK cell function due to its greater selectivity for BTK [40, 41, 53]. HLA-E is also recognised by the activating receptor NKG2C expressed on NK cells and interestingly, NKG2C + NK cells are reduced in CLL patients, with high expression of HLA-E detected on tumour cells [54]. In our study, the downregulation of HLA-E by selinexor increased total NK mediated lysis of CLL cells, indicating that any loss of stimulation via NKG2C:HLA-E interactions is outweighed by the loss of inhibitory signalling via NKG2A. Interestingly, 9/16 CLL samples showed a bimodal downregulation of HLA-E in response to selinexor, perhaps reflecting increased sensitivity of certain CLL sub-populations to HLA-E downregulation.

NKG2A is upregulated on NK cells following expansion using multiple different clinically relevant techniques [55]. This suggests that XPO1 inhibitors could potentially be used to prime B cell malignancies for NK and CAR-NK therapies by abrogating NKG2A-mediated inhibitory signalling. This is important because CAR-NK cells are currently under clinical evaluation in CLL and NHL (NCT03056339, NCT05020678, NCT05410041). NK cell activation was not affected by selinexor in our assays, in accordance with previous reports [8], however XPO1 inhibitors have previously been shown to inhibit T cell activation when given at frequent high doses [56]. The prolonged duration for which HLA-E remains suppressed following selinexor treatment indicates that combination strategies could be designed in which selinexor is given prior to T cell directed therapeutics for maximal therapeutic efficacy. Indeed, recent data showed that the simultaneous administration of selinexor with CAR-T cell therapy was not beneficial in lymphoma models [57], however the sequential administration of selinexor and then CD19 targeted CAR-T cells provided enhanced efficacy compared to selinexor or CAR-T cells alone [58]. NKG2A expression is induced on the surface of CD8 + T cells following multiple cell division cycles [59] and blockade of NKG2A function enhances CD8 + T cell function [60]. HLA-E downregulation by selinexor could therefore provide an explanation for the enhanced activation evident in these studies and warrants further investigation.

In summary, this study identifies that XPO1 inhibition primes CLL cells for NK cell effector functions and that microenvironmental support inhibits NK cell activation against CLL. Selinexor also augmented NK cell activity against CLL cells in the presence of microenvironmental support signals, and in combination with acalabrutinib and anti-CD20 antibodies. XPO1 inhibition may therefore be a promising strategy to enhance the efficacy of NK cell directed therapies for patients with B cell malignancies.

### Supplementary information

Supplementary Table 1

## Data Availability

All data generated or analysed during this study are included in this published article [and its supplementary information files] and the raw data will be made freely available upon request.

## References

[1] Fu SC, Huang HC, Horton P, Juan HF. ValidNESs: a database of validated leucine-rich nuclear export signals. *Nucleic Acids Res.* 2013;41. 10.1093/nar/gks936. 10.1093/nar/gks936 PMC3531083 23093589

[2] Santiago A, Li D, Zhao LY, Godsey A, Liao D (2013). p53 SUMOylation promotes its nuclear export by facilitating its release from the nuclear export receptor CRM1. Mol Biol Cell.

[3] Gravina GL, Senapedis W, McCauley D, Baloglu E, Shacham S, Festuccia C (2014). Nucleo-cytoplasmic transport as a therapeutic target of cancer. J Hematol Oncol.

[4] Gadal O, Strauß D, Kessl J, Trumpower B, Tollervey D, Hurt E (2001). Nuclear export of 60S ribosomal subunits depends on Xpo1p and requires a nuclear export sequence-containing factor, Nmd3p, that associates with the large subunit protein Rpl10p. Mol Cell Biol.

[5] Tabe Y, Kojima K, Yamamoto S, Sekihara K, Matsushita H, Davis RE, et al. Ribosomal biogenesis and translational flux inhibition by the selective inhibitor of nuclear export (sine) XPO1 antagonist KPT-185. *PLoS One.* 2015;10. 10.1371/journal.pone.0137210. 10.1371/journal.pone.0137210 PMC4560410 26340096

[6] Azizian NG, Azizian NG, Li Y, Li Y (2020). XPO1-dependent nuclear export as a target for cancer therapy. J Hematol Oncol.

[7] Walker JS, Hing ZA, Harrington B, Baumhardt J, Ozer HG, Lehman A, et al. Recurrent XPO1 mutations alter pathogenesis of chronic lymphocytic leukemia. *J Hematol Oncol*. 2021;14. 10.1186/s13045-021-01032-2. 10.1186/s13045-021-01032-2 PMC7809770 33451349

[8] Lapalombella R, Sun Q, Williams K, Tangeman L, Jha S, Zhong Y (2012). Selective inhibitors of nuclear export show that CRM1/XPO1 is a target in chronic lymphocytic leukemia. Blood.

[9] Lucas F, Rogers KA, Harrington BK, Pan A, Yu L, Breitbach J (2019). Eμ-TCL1xMyc: a novel mouse model for concurrent CLL and B-cell lymphoma. Clin Cancer Res.

[10] Stephens DM, Huang Y, Ruppert AS, Walker JS, Canfield D, Cempre CB, et al. Selinexor combined with ibrutinib demonstrates tolerability and safety in advanced B-cell malignancies: a phase I study. *Clin Cancer Res*. 2022;28:3242–7. 10.1158/1078-0432.CCR-21-3867 PMC9364840 35608822

[11] Fisher JG, Walker CJ, Doyle AD, Johnson PW, Forconi F, Cragg MS (2021). Selinexor enhances NK cell activation against malignant B cells via downregulation of HLA-E. Front Oncol.

[12] Huntington ND, Cursons J, Rautela J (2020). The cancer–natural killer cell immunity cycle. Nat Rev Cancer..

[13] Forconi F, Moss P (2015). Perturbation of the normal immune system in patients with CLL. Blood.

[14] De Weerdt I, Hofland T, De Boer R, Dobber JA, Dubois J, Van Nieuwenhuize D (2019). Distinct immune composition in lymph node and peripheral blood of CLL patients is reshaped during venetoclax treatment. Blood Adv.

[15] McWilliams EM, Mele JM, Cheney C, Timmerman EA, Fiazuddin F, Strattan EJ, et al. Therapeutic CD94/NKG2A blockade improves natural killer cell dysfunction in chronic lymphocytic leukemia. *Oncoimmunology*. 2016;5. 10.1080/2162402X.2016.1226720. 10.1080/2162402X.2016.1226720 PMC5087289 27853650

[16] Veuillen C, Aurran-Schleinitz T, Castellano R, Rey J, Mallet F, Orlanducci F (2012). Primary B-CLL resistance to NK cell cytotoxicity can be overcome in vitro and in vivo by priming NK cells and monoclonal antibody therapy. J Clin Immunol.

[17] Hilpert J, Grosse-Hovest L, Grünebach F, Buechele C, Nuebling T, Raum T (2012). Comprehensive analysis of NKG2D ligand expression and release in leukemia: implications for NKG2D-mediated NK cell responses. J Immunol.

[18] Reiners KS, Topolar D, Henke A, Simhadri VR, Kessler J, Sauer M (2013). Soluble ligands for NK cell receptors promote evasion of chronic lymphocytic leukemia cells from NK cell anti-tumor activity. Blood.

[19] Yano M, Byrd JC, Muthusamy N (2022). Natural killer cells in chronic lymphocytic leukemia: functional impairment and therapeutic potential. Cancers.

[20] Klanova M, Oestergaard MZ, Trneny M, Hiddemann W, Marcus R, Sehn LH (2019). Prognostic impact of natural killer cell count in follicular lymphoma and diffuse large b-cell lymphoma patients treated with immunochemotherapy. Clin Cancer Res.

[21] Bowles JA, Wang S-Y, Link BK, Allan B, Beuerlein G, Campbell M-A (2006). Anti-CD20 monoclonal antibody with enhanced affinity for CD16 activates NK cells at lower concentrations and more effectively than rituximab. Blood.

[22] Liu E, Marin D, Banerjee P, Macapinlac HA, Thompson P, Basar R (2020). Use of CAR-transduced natural killer cells in CD19-positive lymphoid tumors. N Engl J Med.

[23] Demaria O, Gauthier L, Debroas G, Vivier E (2021). Natural killer cell engagers in cancer immunotherapy: next generation of immuno-oncology treatments. Eur J Immunol.

[24] Chu Y, Lamb M, Cairo MS, Lee DA (2022). The future of natural killer cell immunotherapy for B cell non-hodgkin lymphoma (B Cell NHL). Curr Treat Options Oncol.

[25] Tanaka J, Tanaka N, Wang YH, Mistuhashi K, Ryuzaki M, Iizuka Y, et al. Phase I study of cellular therapy using ex vivo expanded natural killer cells from autologous peripheral blood mononuclear cells combined with rituximab-containing chemotherapy for relapsed CD20-positive malignant lymphoma patients. *Haematologica*. 2020;105. 10.3324/HAEMATOL.2019.226696. 10.3324/haematol.2019.226696 PMC7109741 31399525

[26] Yano M, Sharpe C, Lance JR, Ravikrishnan J, Zapolnik K, Mo X (2022). Evaluation of allogeneic and autologous membrane-bound IL-21-expanded NK cells for chronic lymphocytic leukemia therapy. Blood Adv.

[27] Hallek M, Cheson BD, Catovsky D, Caligaris-Cappio F, Dighiero G, Döhner H (2018). iwCLL guidelines for diagnosis, indications for treatment, response assessment, and supportive management of CLL. Blood.

[28] Chiodin G, Drennan S, Martino EA, Ondrisova L, Henderson I, del Rio L (2022). High surface IgM levels associate with shorter response to ibrutinib and BTK bypass in patients with CLL. Blood Adv.

[29] D’Avola A, Drennan S, Tracy I, Henderson I, Chiecchio L, Larrayoz M (2016). Surface IgM expression and function are associated with clinical behavior, genetic abnormalities, and DNA methylation in CLL. Blood.

[30] Kluckova K, Clear AJ, D’Avola A, Rassenti LZ, Kipps TJ, Gribben JG (2022). B-cell receptor signaling induced metabolic alterations in chronic lymphocytic leukemia can be partially bypassed by TP53 abnormalities. HemaSphere.

[31] Calissano C, Damle RN, Marsilio S, Yan XJ, Yancopoulos S, Hayes G (2011). Intraclonal complexity in chronic lymphocytic leukemia: Fractions enriched in recently born/divided and older/quiescent cells. Mol Med.

[32] Bader JC, Abdul Razak AR, Shacham S, Xu H (2021). Pharmacokinetics of Selinexor: the first-in-class selective inhibitor of nuclear export. Clin Pharmacokinet.

[33] Hamblin TJ, Davis Z, Gardiner A, Oscier DG, Stevenson FK (1999). Unmutated Ig VH genes are associated with a more aggressive form of chronic lymphocytic leukemia. Blood.

[34] Carlsten M, Namazi A, Reger R, Levy E, Berg M, St. Hilaire C (2019). Bortezomib sensitizes multiple myeloma to NK cells via ER-stress-induced suppression of HLA-E and upregulation of DR5. Oncoimmunology.

[35] Yamamoto K, Venida A, Yano J, Biancur DE, Kakiuchi M, Gupta S (2020). Autophagy promotes immune evasion of pancreatic cancer by degrading MHC-I. Nature.

[36] Sen Santara S, Crespo Â, Lee D-J, Jacob Hu J, Zhang Y, Chowdhury S et al. The NK receptor NKp46 recognizes ecto-calreticulin on ER-stressed cells. bioRxiv 2021; 2021.10.31.466654.

[37] Höfle J, Trenkner T, Kleist N, Schwane V, Vollmers S, Barcelona B, et al. Engagement of TRAIL triggers degranulation and IFNγ production in human natural killer cells. *EMBO Rep.* 2022;23. 10.15252/EMBR.202154133. 10.15252/embr.202154133 PMC9346491 35758160

[38] Kohrt HE, Sagiv-Barfi I, Rafiq S, Herman SEM, Butchar JP, Cheney C (2014). Ibrutinib antagonizes rituximab-dependent NK cell-mediated cytotoxicity. Blood.

[39] Flinsenberg TWH, Tromedjo CC, Hu N, Liu Y, Guo Y, Thia KYT (2020). Differential effects of BTK inhibitors ibrutinib and zanubrutinib on NK-cell effector function in patients with mantle cell lymphoma. Haematologica.

[40] Barf T, Covey T, Izumi R, Van De Kar B, Gulrajani M, Van Lith B (2017). Acalabrutinib (ACP-196): a covalent bruton tyrosine kinase inhibitor with a differentiated selectivity and in vivo potency profile. J Pharm Exp Ther.

[41] Rajasekaran N, Sadaram M, Hebb J, Sagiv-Barfi I, Ambulkar S, Rajapaksa A (2014). Three BTK-specific inhibitors, in contrast to ibrutinib, do not antagonize rituximab-dependent NK-cell mediated cytotoxicity. Blood.

[42] Golay J, Ubiali G, Introna M (2017). The specific Bruton tyrosine kinase inhibitor acalabrutinib (ACP-196) shows favorable in vitro activity against chronic lymphocytic leukemia B cells with CD20 antibodies. Haematologica.

[43] Burger JA (2020). Treatment of chronic lymphocytic leukemia. N. Engl J Med.

[44] Hayden RE, Pratt G, Roberts C, Drayson MT, Bunce CM (2012). Treatment of chronic lymphocytic leukemia requires targeting of the protective lymph node environment with novel therapeutic approaches. Leuk Lymphoma.

[45] Aguilar-Hernandez MM, Blunt MD, Dobson R, Yeomans A, Thirdborough S, Larrayoz M (2016). IL-4 enhances expression and function of surface IgM in CLL cells. Blood.

[46] André P, Denis C, Soulas C, Bourbon-Caillet C, Lopez J, Arnoux T (2018). Anti-NKG2A mAb is a checkpoint inhibitor that promotes anti-tumor immunity by unleashing both T and NK cells. Cell.

[47] Hofland T, Endstra S, Gomes CKP, De Boer R, De Weerdt I, Bobkov V, et al. Natural killer cell hypo-responsiveness in chronic lymphocytic leukemia can be circumvented in vitro by adequate activating signaling. *HemaSphere*. 2019;3. 10.1097/HS9.0000000000000308. 10.1097/HS9.0000000000000308 PMC6924557 31976482

[48] Parry HM, Stevens T, Oldreive C, Zadran B, McSkeane T, Rudzki Z (2016). NK cell function is markedly impaired in patients with chronic lymphocytic leukaemia but is preserved in patients with small lymphocytic lymphoma. Oncotarget.

[49] MacFarlane AW, Jillab M, Smith MR, Alpaugh RK, Cole ME, Litwin S, et al. NK cell dysfunction in chronic lymphocytic leukemia is associated with loss of the mature cells expressing inhibitory killer cell Ig-like receptors. *Oncoimmunology.* 2017; 6. 10.1080/2162402X.2017.1330235. 10.1080/2162402X.2017.1330235 PMC5543845 28811973

[50] Ghia P, Strola G, Granziero L, Geuna M, Guida G, Sallusto F (2002). Chronic lymphocytic leukemia B cells are endowed with the capacity to attract CD4+,CD40L+ T cells by producing CCL22. Eur J Immunol.

[51] Dubovsky JA, Beckwith KA, Natarajan G, Woyach JA, Jaglowski S, Zhong Y (2013). Ibrutinib is an irreversible molecular inhibitor of ITK driving a Th1-selective pressure in T lymphocytes. Blood.

[52] Khurana D, Arneson LN, Schoon RA, Dick CJ, Leibson PJ (2007). Differential regulation of human NK cell-mediated cytotoxicity by the tyrosine kinase Itk. J Immunol.

[53] Byrd JC, Harrington B, O’Brien S, Jones JA, Schuh A, Devereux S (2016). Acalabrutinib (ACP-196) in relapsed chronic lymphocytic leukemia. N Engl J Med.

[54] Puiggros A, Blanco G, Muntasell A, Rodríguez-Rivera M, Nonell L, Altadill M (2021). Reduced expansion of CD94/NKG2C+ NK cells in chronic lymphocytic leukemia and CLL-like monoclonal B-cell lymphocytosis is not related to increased human cytomegalovirus seronegativity or NKG2C deletions. Int J Lab Hematol.

[55] Fisher JG, Doyle ADP, Graham LV, Khakoo SI, Blunt MD (2022). Disruption of the NKG2A:HLA-E immune checkpoint axis to enhance NK cell activation against cancer. Vaccines.

[56] Tyler PM, Servos MM, De Vries RC, Klebanov B, Kashyap T, Sacham S (2017). Clinical dosing regimen of selinexor maintains normal immune homeostasis and T-cell effector function in Mice: Implications for combination with immunotherapy. Mol Cancer Ther.

[57] Wang S, Sellner L, Wang L, Sauer T, Neuber B, Gong W (2021). Combining selective inhibitors of nuclear export (SINEs) with chimeric antigen receptor (CAR) T cells for CD19-positive malignancies. Oncol Rep.

[58] Stadel R, Liu R, Landesman Y, Wald D, Hosahalli Vasanna S, de Lima MJG (2022). Sequential administration of selinexor then CD19 CAR-T cells exhibits enhanced efficacy in a mouse model of human non-Hodgkin’s lymphoma. Blood.

[59] Borst L, Sluijter M, Sturm G, Charoentong P, Santegoets SJ, van Gulijk M (2022). NKG2A is a late immune checkpoint on CD8 T cells and marks repeated stimulation and cell division. Int J Cancer.

[60] van Montfoort N, Borst L, Korrer MJ, Sluijter M, Marijt KA, Santegoets SJ (2018). NKG2A blockade potentiates CD8 T cell immunity induced by cancer vaccines. Cell.

