# Supplementary material for: XPO1 inhibition sensitises CLL cells to NK cell mediated cytotoxicity and overcomes HLA-E expression
Source: Leukemia. 2023 Aug 1;37(10):2036–49. doi: 10.1038/s41375-023-01984-z (PMC10539165; doi:10.1038/s41375-023-01984-z)
Supplement: Supplementary file 1 — Supplementary Table 1 [file 41375_2023_1984_MOESM1_ESM.docx]

**Supplementary Table 1. Patient Data.**

NA: Not Available
